# Supplementary material for: Differential Expression of Genes at Panicle Initiation and Grain Filling Stages Implied in Heterosis of Rice Hybrids
Source: Int J Mol Sci. 2020 Feb 6;21(3):1080. doi: 10.3390/ijms21031080 (PMC7038112; doi:10.3390/ijms21031080)
Supplement: Supplementary file 1 [file ijms-21-01080-s001.zip › Additional Figure 1.docx]

**Additional Figure 1:** Hierarchical cluster analysis of all transcripts (a) Ajay hybrid and its parental lines (b) Rajalaxmi hybrid and its parental lines. The color key represents FPKM normalized log_2_ transformed counts. S1 and S2 denote leaf samples from panicle initiation (PI) and grain filling (GF) stage respectively. PK117: IR42266-29-3R; 31A: CRMS31A; 32A: CRMS32A.
