# Supplementary material for: Differential Expression of Genes at Panicle Initiation and Grain Filling Stages Implied in Heterosis of Rice Hybrids
Source: Int J Mol Sci. 2020 Feb 6;21(3):1080. doi: 10.3390/ijms21031080 (PMC7038112; doi:10.3390/ijms21031080)
Supplement: Supplementary file 1 [file ijms-21-01080-s001.zip › Additional file 6.docx]

**Table S6**: Heterosis for agro-morphological characters in two hybrids, Ajay and Rajalaxmi

| S.No. | Traits | CRMS31A  (WA-CMS) | CRMS32A  (Kalinga-I-CMS) | R-42266-29-3R (R-line) | Ajay  (Hybrid) | Rajalaxmi  (Hybrid) | Better parent heterosis (BPH) | |
| --- | --- | --- | --- | --- | --- | --- | --- | --- |
|  |  |  |  |  |  |  | Ajay | Rajalaxmi |
| 1 | Plant type | Semi-dwarf | Semi dwarf | Semi tall | Erect Semi dwarf | Erect Semi dwarf | NA | NA |
| 2 | Leaf length (cm) | 28.5 | 27.0 | 35.0 | 48.5 | 47.5 | 7.38** | 7.02** |
| 3 | Leaf width (cm) | 0.85 | 0.92 | 1.15 | 1.5 | 1.4 | 6.14** | -0.58 |
| 4 | Days to 50% flowering (days) | 100.0 | 104.0 | 95.0 | 100.0 | 106.0 | 0.86 | 2.37** |
| 5 | Panicle exsertion | Exserted | Exserted | Exserted | Fully exserted | Fully exserted | NA | NA |
| 6 | Apiculus colour | Green | Green | Green | Straw | Straw | NA | NA |
| 7 | No. of effective tillers/plant (No.) | 13.0 | 14.0 | 10.0 | 10.0 | 12.0 | -2.50** | -2.22** |
| 8 | No. of panicles/sq.mt (no.) | 380.0 | 390.0 | 300.0 | 255.0 | 278.0 | -1.96* | -4.44** |
| 9 | Plant height ( cm) | 80.0 | 85.0 | 122.5 | 112.0 | 115.0 | 0.81 | 0.63 |
| 10 | Panicle length (cm) | 25.0 | 26.0 | 23.0 | 28.0 | 30.0 | 7.97** | 4.91** |
| 11 | Panicle type | Compact | Compact | Compact | Intermediate | Intermediate | NA | NA |
| 12 | Awning | Absent | Absent | Occasional | Very occasional | Very occasional | NA | NA |
| 13 | Days to maturity (days) | 125.0 | 128.0 | 122.0 | 130.0 | 135.0 | 5.16** | 6.39** |
| 14 | Seed coat (kernel) colour | White | White | Translucent | White | White | NA | NA |
| 15 | Kernel length (mm) | 6.51 | 6.60 | 7.26 | 7.22 | 7.44 | -1.73 | 1.83 |
| 16 | Kernel breadth(mm) | 1.72 | 1.70 | 2.20 | 2.04 | 1.98 | 4.84** | -11.67** |
| 17 | Grain L/B ratio | 3.78 | 3.88 | 3.30 | 4.40 | 4.37 | 9.95** | 14.67** |
| 18 | Kernel appearance | Long slender, Translucent | Long slender, Translucent | Long slender, Translucent | Long slender, translucent | Long slender, translucent | NA | NA |
| 19 | 1,000 grain weight (g) | 18.0 | 19.0 | 25.0 | 23.2 | 22.8 | -2.15 | -6.27** |
| 20 | Grain yield/plant (g) | 13.5 | 14.0 | 19.5 | 24.6 | 25.2 | 19.71** | 32.54** |
| 21 | Hulling recovery (%) | 74.5 | 74.5 | 75.5 | 80.0 | 80.0 | 10.48** | 8.34** |
| 22 | Milling recovery (%) | 65.0 | 65.0 | 65.0 | 72.0 | 73.0 | 7.87** | 9.73** |
| 23 | Head rice recovery (%) | 54.0 | 54.0 | 57.5 | 62.0 | 60.33 | 7.03** | 6.81** |
| 24 | Alkali value | 6.00 | 6.00 | 6.30 | 4.5 | 5.0 | -15.30** | -9.54** |
| 25 | Amylose content (%) | 24.5 | 24.5 | 24.9 | 22.8 | 23.6 | -5.91** | -2.28** |
| 26 | Reaction to abiotic stresses. | Sustain 7-10 days water stagnant | Cold tolerance at seedling stage | Heat tolerant, high pollen load | The hybrid can tolerate excess stagnant water for 10-15 days at vegetative stage. | Tolerance to stagnant excess water for 7 to 10 days at tillering stage. Moderate Cold tolerance at seedling stage | NA | NA |
| 27 | Average yield under normal condition (Yield in kg per ha). | 4500.0 | 5200.0 | 4525.0 | 6000.0 | 6500.0 | 11.36** | 16.19** |

*, ** Significant at 5% and 1% level respectively, NA (Not Applicable): qualitative characters
